# Allelic distribution of *ABO* gene in Chinese centenarians

**DOI:** 10.1002/agm2.12122

**Published:** 2020-09-11

**Authors:** Ying Zhu, Yu Liang, Abdul Haseeb Khan, Minghua Dong, Yiqi Wan, Zhichao Sun, Yi Zeng, Chao Nie, Xiao‐Li Tian

**Affiliations:** ^1^ Human Aging Research Institute (HARI) School of Life Science, and Jiangxi Key Laboratory of Human Aging Nanchang University Nanchang China; ^2^ First Affiliated Hospital of Gannan Medical University Ganzhou China; ^3^ Gannan Medical University Ganzhou China; ^4^ Center for the Study of Aging and Human Development Medical School of Duke University Durham North Carolina USA; ^5^ Center for Healthy Aging and Development Studies National School of Development Peking University Beijing China; ^6^ BGI Shenzhen Shenzhen China; ^7^ BGI Education Center University of Chinese Academy of Sciences Shenzhen China

**Keywords:** *ABO* gene, centenarian, longevity, single nucleotide polymorphisms

## Abstract

**Objective:**

Human ABO blood groups are determined by the alleles *A*, *B*, and *O* (*O01* and *O02*) of the *ABO* gene and have been linked to the risks for cardiovascular diseases and cancers that affect lifespan.

We examined the genetic associations of the *ABO* gene and blood groups with longevity.

**Methods:**

We inspected the frequencies of the *A*, *B*, *O*, and *O02* alleles in a large Chinese centenarian population (n = 2201) and in middle‐aged controls (n = 2330). The single nucleotide polymorphisms were selected as allele *A* (rs507666), *B* (rs8176743, rs8176746, and rs8176749), *O* (rs687289), and *O02* (rs688976, rs549446, and rs512770).

**Results:**

Supported by allelic and genotypic association studies, the frequencies of blood types A, B, O, and AB in centenarian versus control participants were not statistically different: 0.2821 versus 0.2781 (χ^2^ = 0.09, *P* = 0.76), 0.2867 versus 0.3060 (χ^2^ = 2.03, *P* = 0.15), 0.3380 versus 0.3159 (χ^2^ = 2.52, *P* = 0.11), and 0.0859 versus 0.0910 (χ^2^ = 0.37, *P* = 0.54), respectively. Sex had little effect on these distributions.

**Conclusion:**

Integrated with other previous reports, we conclude from this large Chinese cohort that genetic variants of the *ABO* gene and blood groups are not associated with longevity.

## 1 INTRODUCTION

The ABO blood group system was first reported in 1901, based on the presence of A and/or B antigens on the erythrocytic membrane and no corresponding anti‐A and/or B antibodies in serum.^1^ Since then, serological methods have been widely used for blood transfusion in clinical practice. In 1990, Yamamoto cloned the coding cDNA of glycosyltransferase of the *A^1^* (*ABO*) gene,^2^ allowing for the use of biological materials alternative to the blood sample, such as fingernails, hair, saliva, and oral mucous membranes, in the typology of ABO blood groups.

The human *ABO* gene is located at chromosome 9q34.1‐34.2. The main coding regions lie in exon 6 and exon 7. ABO blood groups are decided by alleles *A*, *B*, and *O*, among which alleles *A* and *B* are autosomal dominant. The differences lie in seven nucleotides (c.297A > G, c.526C > G, c.657C > T, c.703G > A, c.796C > A, c.803G > C, and c.930G > A), and allele *O* is a single nucleotide deletion (c.261delG) resulting in a frameshift and early termination with no active enzyme produced. Allele *O* is mainly *O01* and *O02*, which differ in nine nucleotides (c.106G > T, c.188G > A, c.189C > T, c.220C > T, c.297A > G, c.646T > A, c.681G > A, c.771C > T, and c.829G > A).^3^, ^4^, ^5^ The alleles *A* and *B* encode glycosyltransferase to transfer the glycosylates to H substance, forming A and B antigen on erythrocytic membranes,^6^ respectively. With the nonfunctional enzyme, instead of A/B antigen, H antigen is expressed by allele *O*.^7^ ABO phenotypes (commonly referred to as “ABO blood types”) are determined by genotypes, while genotypes *A/A* and *A/O* correspond to phenotype A, *B/B* and *B/O* to phenotype B, *O/O* to phenotype O, and *A/B* to phenotype AB.

The ABO blood group system is the most widely used blood group system.^8^ It not only plays a role in blood transfusion and transplantation but is also of interest to many researchers for its relation with diseases. For instance, it has been shown that blood type A is a risk factor for gastric cancer^9^, ^10^, ^11^ while blood type O is a protective factor for atherosclerosis.^12^, ^13^, ^14^

Cardiovascular diseases and cancers impact the lifespan significantly. Thus, the association between ABO blood types and human longevity has naturally been evaluated previously. It was reported as early as the 1960s that individuals with type A lived longer.^15^ Later, a number of studies reported that type B and O were associated with longer lifespan or longevity phenotypically and genotypically.^16^, ^17^, ^18^, ^19^

However, these findings remain debatable.^20^ The debate is possibly caused by small population sizes and stratifications as well as the moderate effects of blood type on longevity. To our knowledge, the largest population to test the genetic association of blood types with human longevity consisted of only 269 centenarians.^16^ Thus, it has become necessary to evaluate the association of ABO blood groups in large longevity populations.

In order to search for factors that influence healthy aging and longevity, we initiated the Chinese Longitudinal Healthy Longevity Survey (CLHLS) in a large Chinese cohort from 1998 to 2014 and carried out genetic screening, leading to the identification of a number of genes associated with human longevity.^21^, ^22^, ^23^, ^24^ Among these studies, datasets of the genome‐wide association study that included 2178 centenarians and 2299 middle‐aged controls^23^, ^24^ were subjected to searches for the genetic associations of the *ABO* gene and blood groups with longevity.

## 2 METHODS

### 2.1 Samples and data source

Sampling, population quality, and genotyping on the cohort have been reported.^23^, ^24^, ^25^ Samples and data from the CLHLS were randomly selected from half of the counties and cities in 22 of the 31 provinces in China, which means the data cover approximately 85% of the total Chinese population. The study included 2201 centenarians, including 570 males and 1631 females, and a regionally matched control group of 2330 middle‐aged individuals, including 793 males and 1537 females.

### 2.2 Selection of single nucleotide polymorphisms for *ABO* alleles, genotypes, and blood types

Eight single nucleotide polymorphisms (SNPs)—including rs507666 (c.28 + 1179G > A) for allele *A*; rs8176743 (c.703G > A), rs8176746 (c.796C > A), and rs8176749 (c.930G > A) for allele *B*; rs687289 (c.99‐329A > G) for allele *O*; and rs688976 (c.106G > T), rs549446 (c.188G > A), and rs512770 (c.220C > T) for allele *O02* ^4^, ^5^, ^26^—were selected for this study. Individuals with the *O* allele but not the *O02* allele were considered for the *O01* allele.

The frequencies of four alleles (*A*, *B*, *O*, and *O02*), 10 genotypes (*A/A*, *A/O01*, *A/O02*, *B/B*, *B/O01*, *B/O02*, *O01/O01*, *O01/O02*, *O02/O02*, and *A/B*), and four blood types (A, B, O, and AB) were evaluated in 4494 individuals. (The identification of the *ABO* alleles and genotypes are listed in the Supplementary Material).

### 2.3 Statistical analysis

The frequency of each SNP was calculated and used to evaluate its departure from Hardy‐Weinberg equilibrium by a chi‐square test. Differences in allele, genotype, and blood type distribution between cases (centenarians) and controls (middle‐aged individuals) were analyzed using binary logistic regression adjusted for nongenetic covariates under various genetic models.^21^ Sex was also analyzed separately. The chi‐square test was performed using GraphPad Prism (Version 8.4.2). A Bonferroni method was used for multiple comparison correction.^27^ The chi‐square values, odds ratios (ORs), 95% confidence intervals (CIs), and *P* values were presented for all association tests. A *P* value < 0.05 was considered to be statistically significant.

### 2.4 Meta‐analysis

#### 2.4.1 Search strategy

We performed a systematic literature search using PubMed, ScienceDirect, Wiley, Oxford Academic, Web of Science, and SinoMed for studies reporting the association between ABO blood groups and longevity, including relevant articles and reviews (up to April 2020). Only studies published in English were considered. Two search themes were combined using the Boolean operator “AND”; the first theme was “ABO AND longevity,” and the second theme was “ABO AND lifespan.”

#### 2.4.2 Selection criteria

The literature eligibility was evaluated by two investigators (Y. Z. and A. H. K.) independently, and disagreements were resolved by another investigator (Y. L.). Articles were included if: (a) the authors had presented an original, peer‐reviewed study (eg, not a meeting report); (b) the study was a case‐control study or a cohort study; (c) age over 90 years was considered as the case group; (d) the authors had provided ORs and 95% CIs for A versus non‐A, B versus non‐B, O versus non‐O, and AB versus non‐AB, or enough data to calculate them. If there was overlap among the data, we chose the report with more extensive coverage.

#### 2.4.3 Data extraction and study quality assessment

The following information was extracted from the selected studies: the first author’s name, publication year, country, exposure measures, number of participants, case numbers, and control numbers. The quality of the studies was assessed with the Newcastle‐Ottawa Scale (NOS). With a score ranging from 0 to 9, a score ≥7 indicated a high‐quality study.

#### 2.4.4 Data synthesis

Heterogeneity test was conducted before meta‐analysis. Cochran Q and *I* ^2^ statistics were used to evaluate heterogeneity. An *I* ^2^ > 50% was considered to have severe heterogeneity.^28^ If *I* ^2^ > 50%, a random effect model was used to combine study individual effect estimates accounting for heterogeneity. Otherwise, the fixed‐effect model was selected.^29^ A comprehensive meta‐analysis was performed (Stata 14.0) to analyze the overall ORs and 95% CIs for the association between ABO blood types and longevity.

## 3 RESULTS

### 3.1 Allelic association analysis

To determine the *ABO* allelic association with longevity, eight SNPs (rs507666 for allele *A*; rs8176743, rs8176746, and rs8176749 for allele *B*; rs687289 for allele *O*; and rs688976, rs549446, and rs512770 for *O02*) were selected. There was no significant deviation for eight tagging SNPs of the *ABO* gene in the Hardy‐Weinberg equilibrium test for either the case or control groups. Allele frequencies of the *ABO* gene in centenarians and middle‐aged controls were evaluated in the total population as well as in sex‐classified populations (Supplementary Material). No significant difference was found in *ABO* allele distributions in centenarians compared to middle‐aged controls regardless of sex (Table 1).

**Table 1:** The frequencies of *ABO* alleles

Allele	SNP	Group	CN	CF	MN	MF	χ^2^	OR	95% CI	*P* value
*A*	rs507666	Total	902	0.2049	980	0.2103	0.40	0.97	0.87‐1.07	0.53
		Male	223	0.1956	356	0.2245	3.30	0.84	0.70‐1.01	0.07
		Female	679	0.2082	624	0.2030	0.26	1.03	0.91‐1.17	0.61
*B*	rs8176743	Total	899	0.2042	1016	0.2180	2.59	0.92	0.83‐1.02	0.11
		Male	241	0.2114	341	0.2150	0.05	0.98	0.81‐1.18	0.82
		Female	658	0.2017	675	0.2196	3.04	0.90	0.80‐1.01	0.08
*B*	rs8176746	Total	918	0.2085	1039	0.2230	2.78	0.92	0.83‐1.02	0.10
		Male	244	0.2140	345	0.2175	0.05	0.98	0.81‐1.18	0.83
		Female	674	0.2066	694	0.2258	3.43	0.89	0.79‐1.01	0.06
*B*	rs8176749	Total	916	0.2081	1032	0.2215	2.40	0.92	0.84‐1.02	0.12
		Male	244	0.2140	344	0.2169	0.03	0.98	0.82‐1.18	0.86
		Female	672	0.2060	688	0.2238	2.98	0.90	0.80‐1.01	0.08
*O*	rs687289	Total	2576	0.5852	2646	0.5678	2.80	1.07	0.99‐1.17	0.09
		Male	674	0.5912	888	0.5599	2.66	1.14	0.97‐1.33	0.10
		Female	1902	0.5831	1758	0.5719	0.81	1.05	0.96‐1.16	0.37
*O02*	rs512770	Total	1103	0.2506	1156	0.2481	0.08	1.01	0.46‐1.25	0.78
		Male	294	0.2579	381	0.2402	1.11	1.10	0.92‐1.31	0.29
		Female	809	0.2480	775	0.2521	0.14	0.98	0.87‐1.10	0.71
*O02*	rs688976	Total	1103	0.2506	1156	0.2481	0.08	1.01	0.46‐1.25	0.78
		Male	294	0.2579	382	0.2409	1.03	1.10	0.92‐1.31	0.31
		Female	809	0.2480	774	0.2518	0.12	0.98	0.87‐1.10	0.73
*O02*	rs549446	Total	1104	0.2508	1157	0.2483	0.08	1.01	0.46‐1.12	0.78
		Male	294	0.2579	383	0.2415	0.96	1.09	0.92‐1.30	0.33
		Female	810	0.2483	774	0.2518	0.10	0.98	0.88‐1.10	0.75

Abbreviations: CF, frequency in centenarians; CI, confidence interval; CN, number of centenarians; MF, frequency in middle‐aged controls; MN, number of middle‐aged controls; OR, odds ratio; SNP, single nucleotide polymorphism.

### 3.2 Genotypic association analysis

A comprehensive analysis of these eight SNPs was executed to determine the *ABO* genotypic association with longevity. We divided *ABO* genotypes into 10 groups (*A/A*, *A/O01*, *A/O02*, *B/B*, *B/O01*, *B/O02*, *O01/O01*, *O01/O02*, *O02/O02*, and *A/B*), and calculated their frequencies separately. The results suggested no significant difference in *ABO* genotypes in centenarians compared to middle‐aged controls after Bonferroni correction regardless of the sex (Table 2).

**Table 2:** The frequencies of *ABO* genotypes

Genotype	Group	CN	CF	MN	MF	χ^2^	OR	95% CI	*P* value	*P*_corr_
*A/A*	Total	87	0.0395	105	0.0451	0.86	0.87	0.65‐1.17	0.36	NT
	Male	19	0.0333	42	0.0530	2.99	0.62	0.36‐1.07	0.08	NT
	Female	68	0.0417	63	0.0410	0.01	1.02	0.72‐1.46	0.92	NT
*A/O01*	Total	290	0.1318	313	0.1343	0.07	0.98	0.82‐1.16	0.80	NT
	Male	72	0.1263	108	0.1362	0.28	0.92	0.67‐1.25	0.60	NT
	Female	218	0.1337	205	0.1334	0.00	1.00	0.82‐1.23	0.97	NT
*A/O02*	Total	244	0.1109	230	0.0987	1.78	1.13	0.94‐1.38	0.18	NT
	Male	61	0.1070	85	0.1072	0.00	1.00	0.71‐1.40	1.00	NT
	Female	183	0.1122	145	0.0943	2.72	1.21	0.96‐1.53	0.10	NT
*B/B*	Total	93	0.0423	107	0.0459	0.36	0.92	0.69‐1.22	0.55	NT
	Male	26	0.0456	44	0.0555	0.66	0.81	0.50‐1.33	0.42	NT
	Female	67	0.0411	63	0.0410	0.00	1.00	0.70‐1.41	1.00	NT
*B/O01*	Total	319	0.1449	329	0.1412	0.13	1.03	0.87‐1.22	0.72	NT
	Male	85	0.1491	102	0.1286	1.18	1.19	0.87‐1.62	0.28	NT
	Female	234	0.1435	227	0.1477	0.11	0.97	0.79‐1.18	0.74	NT
*B/O02*	Total	219	0.0995	277	0.1189	4.36	0.82	0.68‐0.99	0.04	
	Male	56	0.0982	81	0.1021	0.06	0.96	0.66‐1.38	0.81	NT
	Female	163	0.0999	196	0.1275	5.99	0.76	0.61‐0.95	0.01	
*O01/O01*	Total	246	0.1118	236	0.1013	1.31	1.12	0.92‐1.34	0.25	NT
	Male	68	0.1193	88	0.1110	0.23	1.08	0.78‐1.52	0.63	NT
	Female	178	0.1091	148	0.0963	1.41	1.15	0.91‐1.45	0.23	NT
*O01/O02*	Total	360	0.1636	357	0.1532	0.91	1.08	0.92‐1.27	0.34	NT
	Male	85	0.1491	110	0.1387	0.29	1.09	0.80‐1.47	0.59	NT
	Female	275	0.1686	247	0.1607	0.36	1.06	0.88‐1.28	0.55	NT
*O02/O02*	Total	138	0.0627	143	0.0614	0.03	1.02	0.80‐1.30	0.85	NT
	Male	45	0.0789	51	0.0643	1.09	1.25	0.82‐1.87	0.30	NT
	Female	93	0.0570	92	0.0599	0.12	0.95	0.70‐1.28	0.73	NT
*A/B*	Total	189	0.0859	212	0.0910	0.37	0.94	0.76‐1.15	0.54	NT
	Male	49	0.0860	71	0.0895	0.05	0.96	0.66‐1.39	0.82	NT
	Female	140	0.0858	141	0.0917	0.34	0.93	0.73‐1.18	0.56	NT

Abbreviations: CF, frequency in centenarians; CI, confidence interval; CN, number of centenarians; MF, frequency in middle‐aged controls; MN, number of middle‐aged controls; NT, not tested; OR, odds ratio; *P*_corr_, *P* value after Bonferroni correction.

### 3.3 Phenotypic association analysis

We incorporated genotypes that represent the same phenotypes—that is, genotypes *A/A*, *A/O01*, and *A/O02* for blood type A; genotypes *B/B*, *B/O01*, and *B/O02* for blood type B; genotypes *O01/O01*, *O01/O02*, and *O02/O02* for blood type O; and genotype *A/B* for blood type AB—to determine the association between ABO blood types and longevity. According to the results from the chi‐square test, no significant difference was found in centenarians compared to middle‐aged controls in ABO blood types after Bonferroni correction regardless of the sex (Table 3).

**Table 3:** The frequencies of ABO blood types

Blood type	Group	CN	CF	MN	MF	χ^2^	OR	95% CI	*P* value	*P*_corr_
A	Total	621	0.2821	648	0.2781	0.09	1.02	0.90‐1.16	0.76	NT
	Male	152	0.2667	235	0.2963	1.44	0.86	0.68‐1.10	0.23	NT
	Female	469	0.2876	413	0.2687	1.40	1.10	0.94‐1.28	0.24	NT
B	Total	631	0.2867	713	0.3060	2.03	0.91	0.80‐1.04	0.15	NT
	Male	167	0.2930	227	0.2863	0.07	1.03	0.82‐1.31	0.79	NT
	Female	464	0.2845	486	0.3162	3.79	0.86	0.74‐1.00	0.05	
O	Total	744	0.3380	736	0.3159	2.52	1.11	0.98‐1.25	0.11	NT
	Male	198	0.3474	249	0.3140	1.68	1.16	0.93‐1.46	0.20	NT
	Female	546	0.3348	487	0.3169	1.16	1.09	0.93‐1.26	0.28	NT
AB	Total	189	0.0859	212	0.0910	0.37	0.94	0.76‐1.15	0.54	NT
	Male	49	0.0860	71	0.0895	0.05	0.96	0.66‐1.39	0.82	NT
	Female	140	0.0858	141	0.0917	0.34	0.93	0.73‐1.18	0.56	NT

Abbreviations: CF, frequency in centenarians; CI, confidence interval; CN, number of centenarians; MF, frequency in middle‐aged controls; MN, number of middle‐aged controls; NT, not tested; OR, odds ratio; *P*_corr_, *P* value after Bonferroni correction.

### 3.4 Meta‐analysis

To compare our findings with the previously published studies, we performed a meta‐analysis.

#### 3.4.1 Literature search

**Figure 1:**
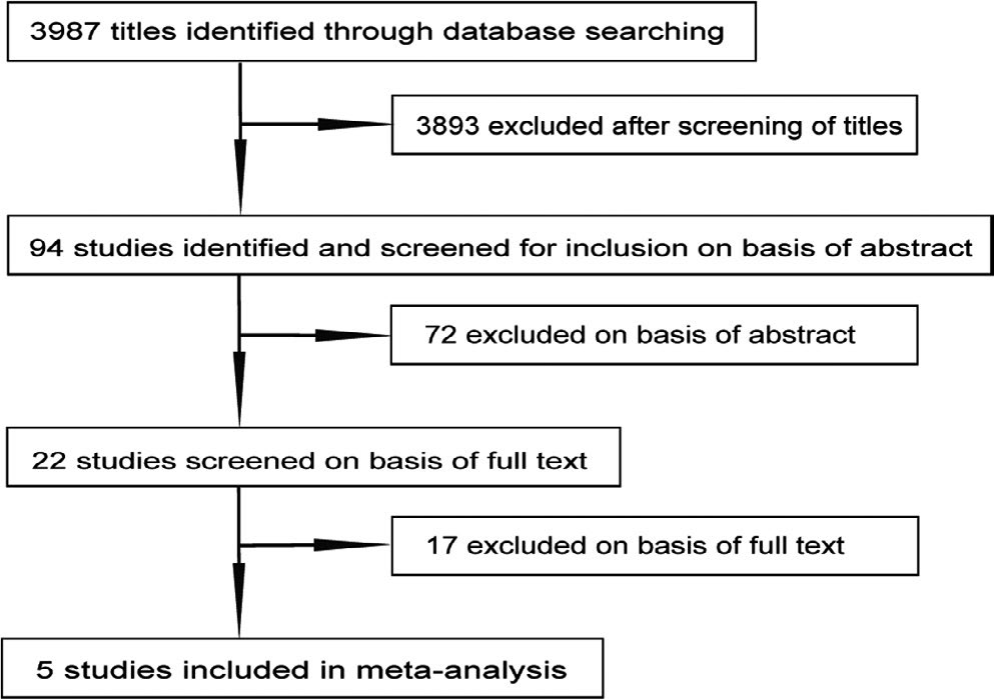
Flowchart representing the study selection process for the meta‐analysis.

Using the search strategy, 3987 citations were identified. After screening based on titles and abstracts, 22 citations remained for further full‐text review. Finally, based on the selection criteria, only five studies were included^16^, ^18^, ^30^, ^31^, ^32^ (Figure 1).

#### 3.4.2 Data extraction and study quality assessment

All of these five articles were case‐control studies with an NOS score ≥ 7. One study only analyzed blood type O and non‐O. First author name, publication year, country, exposure measures, number of centenarians, case numbers, control numbers, and NOS scores are listed in Table 4. Data from the current study are also listed.

**Table 4:** Characteristics, ABO blood types distribution, and NOS scores of included studies

First Author, Y^Ref.^		Sturgeon, 1969^30^	Coppola, 2003^31^	Shimizu, 2004^16^	Mengoli, 2015^32^	Franchini, 2016^18^	Current study, 2020
Country		Turkey	Italy	Japan	Italy	Italy	China
Exposure measures		Serological methods	Blood test	Blood test	Electronic clinical records	Standard micro‐column agglutination technology	Genotyping by SNPs
Number of centenarians		50	74	269	252	165	2201
A/non‐A	Cases	22/28		92/177	108/144	55/110	621/1580
	Controls	47/63		2759/4394	2145/2880	2086/2977	648/1682
B/non‐B	Cases	11/39		79/190	22/230	14/151	631/1570
	Controls	20/90		1570/5583	575/4450	541/4522	713/1617
O/non‐O	Cases	11/39	32/42	76/193	110/142	93/72	744/1457
	Controls	37/73	39/71	2153/5000	2087/2938	2201/2862	736/1594
AB/non‐AB	Cases	6/44		22/247	12/240	3/162	189/2012
	Controls	6/104		671/6482	218/4807	235/4828	212/2118
NOS	7	8	8	8	7	8	

Abbreviations: NOS, Newcastle‐Ottawa Scale; SNPs, single nucleotide polymorphisms.

#### 3.4.3 Association between ABO blood types and longevity by meta‐analysis

**Figure 2:**
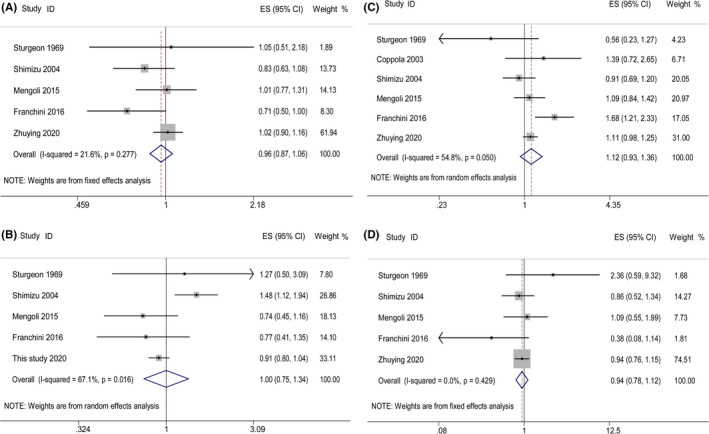
Forest plots of ABO blood types and longevity: (A) blood type A and longevity, (B) blood type B and longevity, (C) blood type O and longevity, (D) blood type AB and longevity.

Since *I* ^2^ > 50% in blood type B versus non‐B and O versus non‐O groups, the random effect model was used. For blood type A versus non‐A and AB versus non‐AB, the fixed‐effect model was used. No statistically significant difference between ABO blood types and longevity was shown by forest plots (Figure 2).

## 4 DISCUSSION

In this study, we evaluated the association of *ABO* alleles, genotypes, and blood types with longevity in our large Chinese centenarian population as well as in previously published datasets and found that genetic variants of *ABO* genes are not associated with the human longevity trait.

Longevity is a complex trait that is affected by both genetic and environmental factors, including diseases and personalities.^33^, ^34^, ^35^, ^36^ Over the past decades, huge efforts have been made to evaluate the genetic contribution to human longevity, leading to the identification of several genes or loci associated with centenarians or exceptionally long‐lived individuals through a candidate gene approach or genome‐wide association study.^21^, ^22^, ^23^, ^37^, ^38^ Human ABO blood groups are genetically determined and have been shown to influence diseases and personalities^39^; therefore, they could possibly influence lifespan, including longevity.

Our study does not support the genetic association of *ABO* alleles with human longevity, as no statistical differences were found between centenarians and middle‐aged controls even without multiple comparison correction, which is in agreement with the previous studies.^20^, ^30^ Our meta‐analysis showed a consistent conclusion.

We carefully reviewed the studies that had previously reported a positive association between ABO blood groups and longevity.^15^, ^16^, ^17^, ^20^, ^30^ As listed in Table 5, small sample size and statement for longevity are two common problems, which are critical influencers for population‐based studies as the population is heterogeneous and stratified by many hidden or unnoticed factors. In this view, a larger population should produce a more robust statistical estimation. In this study, 2201 centenarians and 2330 regionally matched middle‐aged individuals as controls were included, presenting the largest population ever for estimating the association between ABO and longevity and providing sufficient statistical power for the statement.

**Table 5:** Summary of longevity with ABO blood group association

First author, Year^Ref.^	Population source	Case number/Mean age (y)	Control number/Mean age (y)	*P* value	Conclusion
Murray, 1961^15^	United Kingdom	281/71.7	352/75.8	<0.025	A decrease in blood group O and an increase in group A in the healthy group compared with the geriatric group.
Sturgeon, 1969^30^	Turkey	50/105	110/36	>0.05	No association between ABO blood groups and longevity was found.
Shimizu, 2004^16^	Japan	269/101.2	7153/54.8	0.04	Blood type B individuals might live longer.
Vasto, 2011^20^	Italy	38/(100‐107)	59/(45‐65)	>0.05	No association between ABO blood groups and longevity was found.
Brecher and Hay, 2011^17^	United States	772 death patients/(0‐97)		<0.01	Instead of a marker for longevity, blood type B might be a marker for earlier death.

The current study does not debate the association between ABO blood groups and specific diseases and risks, such as myocardial infarction and coronary artery disease,^13^, ^40^, ^41^ ischemic stroke,^42^ or cancers,^9^, ^43^ as previously reported (Table 6). Centenarians represent a model of human healthy aging in contrast to those who suffer from diseases or even death after middle age. That *ABO* genotypes are associated with diseases, even life‐threatening diseases, but not with longevity may imply a notion that disease and longevity are two biological processes with distinct pathways. On the other hand, the two biological processes also share some common pathways. For example, we have shown in our previous study that immune response and inflammation, mitogen‐activated protein kinase, sucrose, and xenobiotic metabolism significantly contribute to longevity,^23^ but these have also been linked to various diseases and the aging process. Longevity is a multifactorial and polygenic trait, and it has a group of influencers, including risks and diseases, which are intermediate phenotypes that contribute to the longevity trait in a more complicated way than expected.

**Table 6:** Summary of subjects with *ABO* allelic and/or genotypic association

First author, Year^Ref.^	Method	Associated allele or genotype	Subject	Population source	Case number/Mean age (y)	*P* value	Conclusion
Barbalic, 2010^41^	GWAS	*A^1^*	sICAM‐1	ARIC,^a^ CHS,^b^ FHS,^c^ RS^d^	829/55.8,^a^ 6845/49.4,^b^ 600/>70.3,^c^ 1487/72.8^d^	9.9E‐07	Both sP‐selectin and sICAM‐1 were associated with *A^1^* allele of ABO blood group (negative correlation).
Barbalic, 2010^41^	GWAS	*A^1^*	sP‐selectin	ARIC,^a^ FHS,^c^ RS^d^	673/56.6,^a^ 3036/>61.0,^c^ 406/69.4^d^	1.8E‐11	
de Paula Sabino, 2014^42^	Case study	*O01*	IS	HUUH	86/36	<0.001	*O01* allele was an independent variable for IS patients.
Tirado, 2005^44^	Case study	*A^1^* and non‐O	VTE	THU	250/47.6	<0.001	Non‐O blood groups, especially with *A^1^* allele, were independent risk factors for VTE.
Nakao, 2011^9^	Case study	Allele *A* and *B*, genotype *A/A*, *A/B*, *A/O*, *O/O*, *B/O*, *B/B*	GC	ACCH	703/(20‐79)	*A^1^*: <0.001, *B*: 0.071	Allele *A* and *B* were associated with increased and decreased risk of GC, respectively, and the *ABO* genotypic rank of GC was: *A/A* > *A/B* > *A/O* > *O/O* > *B/O* > *B/B*.
Souto, 2000^45^	Combined linkage and association test	*O/O* and non‐*O/O*	vWF and FVIII	GAIT Project	397/37.7	vWF:1E‐7, FVIII: 8.2E‐6	*ABO* locus had a functional effect on vWF and factor VIII. The rank of vWF and FVIII levels in *ABO* genotypes was: *A/B* > *A/A* > *A/O* > *B/O* > *O/O*.
Melzer, 2008^46^	GWAS	*O/O* and non‐*O/O*	TNF‐alpha	InCHIANTI	1200/68.4	6.80E‐40	An assay‐specific association appeared between ABO blood group and TNF‐alpha levels.
Paterson, 2009^47^	GWAS	*O/O* and non‐*O/O*	sE‐selectin	DCCT and EDIC	685/51.8	3.7E‐29	*ABO* was a major locus for sE‐selectin levels. The rank of sE‐selectin level in *ABO* genotypes was: *O/O*≈*A^2^/O* > *B/O*≈*B/B* > *A^2^/B* > *A^1^/B*≈*A^1^/O* > *A^1^/A^2^* > *A^1^/A^1^*.
Antwi, 2018^43^	Pooled analysis	*O/O* and non‐*O/O*	PC	PanC4,^e^ PanScan^f^	2414/65.1,^e^ 1268/67.2^f^	< 0.0001,^e^ 0.002^f^	Genotype‐derived non‐O blood type was associated with increased pancreatic cancer risk.

Abbreviations: ACCH, Aichi Cancer Center Hospital (Nagoya, Japan); ARIC, Atherosclerosis Risk in Communities; CHS, Cardiovascular Health Study; DCCT, Diabetes Control and Complications Trial; EDIC, Epidemiology of Diabetes Intervention and Complications; FHS, Framingham Heart Study; FVIII, factor VIII; GAIT, Genetic Analysis of Idiopathic Thrombophilia; GC, gastric cancer; GWAS, genome‐wide association study; HUUH, Hematology Unit of the University Hospital (Federal University of Minas Gerais, Belo Horizonte MG); InCHIANTI, A population‐based study of persons living in the Chianti geographic area (Tuscany, Italy); IS, ischemic stroke; PanC4, Pancreatic Cancer Case‐Control Consortium; PanScan, Pancreatic Cancer Cohort Consortium; PC, pancreatic cancer; RS, Rotterdam Study; sE‐selectin, soluble E‐selectin; sICAM‐1, soluble intercellular adhesion molecule‐1; sP‐selectin, soluble P‐selectin; THU, thrombosis and hemostasis units from local hospitals (Spain); TNF‐alpha, tumor necrosis factor‐alpha; VTE, venous thromboembolism; vWF, von Willebrand factor. The superscript symbols a‐f in the Population source column correspond to those in the next column.

Since all of our subjects are Han Chinese, our study has a population limitation. It is necessary to validate our findings in other populations. In summary, our study shows that genetic variants of the *ABO* gene are not associated with the human longevity trait.

## AUTHOR CONTRIBUTIONS

Conceptualization, resources, supervision, and funding acquisition for this study: Xiao‐Li Tian; data analyses: Ying Zhu, Yiqi Wan, Yu Liang, Minghua Dong, and Zhichao Sun; writing: Ying Zhu (original draft), Xiao‐Li Tian and Abdul Haseeb Khan (revised manuscript); genome‐wide association study of Chinese centenarians: Yi Zeng, Chao Nie, and Xiao‐Li Tian.

**CONFLICTS OF INTEREST**

Nothing to disclose.

## Supporting information

Supplementary Material
