# Supplementary material for: Allelic distribution of ABO gene in Chinese centenarians
Source: Aging Med (Milton). 2020 Sep 11;3(3):195–204. doi: 10.1002/agm2.12122 (PMC7574633; doi:10.1002/agm2.12122)
Supplement: Supplementary file 1 — Supplementary Material [file AGM2-3-195-s001.pdf]

Allelic Distribution of *ABO* gene in Chinese Centenarians

Ying Zhu[1,2] | Yu Liang[1] | Abdul Haseeb Khan[1] | Minghua Dong[3] | Yiqi Wan[1] | Zhichao Sun[1] | Yi Zeng[4,5] | Chao Nie[6,7] | Xiao-Li Tian[1]

[1] Human Aging Research Institute (HARI), School of Life Science, and Jiangxi Key Laboratory of Human Aging, Nanchang University, Nanchang, Jiangxi, China

[2] First Affiliated Hospital of Gannan Medical University, Ganzhou, Jiangxi, China

[3] Gannan Medical University, Ganzhou, Jiangxi, China

[4] Center for the Study of Aging and Human Development, Medical School of Duke University, Durham, NC, USA

[5] Center for Healthy Aging and Development Studies, National School of Development, Peking University, Beijing, China

[6] BGI Shenzhen, Shenzhen, China

[7] BGI Education Center, University of Chinese Academy of Sciences, Shenzhen, China

Correspondence

Xiao-Li Tian, Human Population Genetics, A217 Life Science Building, Human Aging Research Institute and School of Life Science, Nanchang University, 999 Xuefu Str., Honggutan New District, Nanchang city, Jiangxi Province 330031, China.

Email: tianxiaoli@ncu.edu.cn

SUPPLEMENTARY:

SUPPLEMENTARY TABLE 1 Eight SNPs comprehensive analysis

number	sex (1=male; 2=female)	1 (middle-aged)/ 2 (centenarians)	rs507666 (*A*)	rs8176746 (*B*)	rs8176743 (*B*)	rs8176749 (*B*)	rs687289 (*O*)	rs512770 (*O02*)	rs688976 (*O02*)	rs549446 (*O02*)	*genotype*	phenotype
1	1	2	AA	CC	00	GG	TT	GG	GG	00	*A/A*	A
18	1	2	AA	CC	GG	GG	TT	GG	GG	CC	*A/A*	A
3	2	2	AA	CC	00	GG	TT	GG	GG	CC	*A/A*	A
2	2	2	AA	CC	GG	GG	TT	GG	GG	00	*A/A*	A
63	2	2	AA	CC	GG	GG	TT	GG	GG	CC	*A/A*	A
1	1	1	AA	CC	00	GG	TT	GG	GG	CC	*A/A*	A
41	1	1	AA	CC	GG	GG	TT	GG	GG	CC	*A/A*	A
2	2	1	AA	CC	00	GG	TT	GG	GG	CC	*A/A*	A
1	2	1	AA	CC	GG	GG	TT	GG	GG	00	*A/A*	A
60	2	1	AA	CC	GG	GG	TT	GG	GG	CC	*A/A*	A
1	2	2	AG	AC	AG	AG	TC	GG	GG	CC	*A/B*	AB
5	1	2	AG	AC	AG	AG	TT	GG	GG	00	*A/B*	AB
44	1	2	AG	AC	AG	AG	TT	GG	GG	CC	*A/B*	AB
1	2	2	AG	AC	AG	00	TT	00	GG	00	*A/B*	AB
2	2	2	AG	AC	AG	AG	TT	00	GG	CC	*A/B*	AB
1	2	2	AG	AC	00	AG	TT	GG	GG	CC	*A/B*	AB
14	2	2	AG	AC	AG	AG	TT	GG	GG	00	*A/B*	AB
120	2	2	AG	AC	AG	AG	TT	GG	GG	CC	*A/B*	AB
1	2	2	AG	AC	AG	GG	TT	GG	GG	CC	*A/B*	AB

1	1	1	AG	AC	AG	AG	TT	00	GG	CC	*A/B*	AB
4	1	1	AG	AC	AG	AG	TT	GG	GG	00	*A/B*	AB
66	1	1	AG	AC	AG	AG	TT	GG	GG	CC	*A/B*	AB
3	2	1	AG	AC	00	AG	TT	GG	GG	CC	*A/B*	AB
3	2	1	AG	AC	AG	AG	TT	GG	GG	00	*A/B*	AB
1	2	1	AG	AC	AG	AG	TT	GG	00	CC	*A/B*	AB
132	2	1	AG	AC	AG	AG	TT	GG	GG	CC	*A/B*	AB
2	2	1	AG	AC	AG	GG	TT	GG	GG	CC	*A/B*	AB
1	1	2	AG	CC	GG	GG	TC	00	GG	CC	*A/O01*	A
1	1	2	AG	CC	00	GG	TC	GG	GG	00	*A/O01*	A
1	1	2	AG	CC	00	GG	TC	GG	GG	CC	*A/O01*	A
3	1	2	AG	CC	GG	GG	TC	GG	GG	00	*A/O01*	A
66	1	2	AG	CC	GG	GG	TC	GG	GG	CC	*A/O01*	A
1	2	2	AG	CC	GG	GG	TC	00	GG	00	*A/O01*	A
1	2	2	AG	CC	GG	00	TC	GG	GG	CC	*A/O01*	A
1	2	2	AG	CC	GG	AG	TC	GG	GG	CC	*A/O01*	A
2	2	2	AG	CC	00	GG	TC	GG	GG	00	*A/O01*	A
15	2	2	AG	CC	00	GG	TC	GG	GG	CC	*A/O01*	A
9	2	2	AG	CC	GG	GG	TC	GG	GG	00	*A/O01*	A
189	2	2	AG	CC	GG	GG	TC	GG	GG	CC	*A/O01*	A
3	1	1	AG	CC	00	GG	TC	GG	GG	CC	*A/O01*	A
105	1	1	AG	CC	GG	GG	TC	GG	GG	CC	*A/O01*	A
1	2	1	AG	CC	GG	GG	TC	00	GG	CC	*A/O01*	A
2	2	1	AG	CC	00	GG	TC	GG	GG	CC	*A/O01*	A
5	2	1	AG	CC	GG	GG	TC	GG	GG	00	*A/O01*	A
197	2	1	AG	CC	GG	GG	TC	GG	GG	CC	*A/O01*	A

1	1	2	AG	CC	GG	GG	CC	AG	TG	TC	*A/O02*	A
1	2	2	AG	CC	GG	GG	CC	AG	TG	TC	*A/O02*	A
2	2	1	AG	CC	GG	GG	CC	AG	TG	TC	*A/O02*	A
5	1	2	AG	CC	00	GG	TC	AG	TG	TC	*A/O02*	A
55	1	2	AG	CC	GG	GG	TC	AG	TG	TC	*A/O02*	A
12	2	2	AG	CC	00	GG	TC	AG	TG	TC	*A/O02*	A
170	2	2	AG	CC	GG	GG	TC	AG	TG	TC	*A/O02*	A
1	1	1	AG	CC	GG	GG	TC	00	TG	TC	*A/O02*	A
3	1	1	AG	CC	00	GG	TC	AG	TG	TC	*A/O02*	A
1	1	1	AG	CC	GG	GG	TC	AG	GG	CC	*A/O02*	A
1	1	1	AG	CC	GG	GG	TC	AG	GG	TC	*A/O02*	A
79	1	1	AG	CC	GG	GG	TC	AG	TG	TC	*A/O02*	A
6	2	1	AG	CC	00	GG	TC	AG	TG	TC	*A/O02*	A
137	2	1	AG	CC	GG	GG	TC	AG	TG	TC	*A/O02*	A
1	1	2	GG	AA	AA	AA	TT	GG	GG	00	*B/B*	B
25	1	2	GG	AA	AA	AA	TT	GG	GG	CC	*B/B*	B
1	2	2	GG	AA	00	AA	TT	GG	GG	CC	*B/B*	B
4	2	2	GG	AA	AA	AA	TT	GG	GG	00	*B/B*	B
1	2	2	GG	AA	AA	AA	TC	GG	GG	CC	*B/B*	B
61	2	2	GG	AA	AA	AA	TT	GG	GG	CC	*B/B*	B
1	1	1	GG	AA	AA	AA	TT	00	GG	CC	*B/B*	B
1	1	1	GG	AA	00	AA	TT	GG	GG	CC	*B/B*	B
1	1	1	GG	AA	AA	AA	TT	GG	GG	00	*B/B*	B
40	1	1	GG	AA	AA	AA	TT	GG	GG	CC	*B/B*	B
1	1	1	GG	AA	AA	AG	TT	GG	GG	CC	*B/B*	B
1	2	1	GG	AA	00	AA	TT	GG	GG	00	*B/B*	B

1	2	1	GG	AA	00	AA	TT	GG	GG	CC	*B/B*	B
2	2	1	GG	AA	AA	AA	TT	GG	GG	00	*B/B*	B
58	2	1	GG	AA	AA	AA	TT	GG	GG	CC	*B/B*	B
1	2	1	GG	AA	AA	AG	TT	GG	GG	CC	*B/B*	B
2	1	2	GG	AC	00	AG	TC	GG	GG	CC	*B/O01*	B
7	1	2	GG	AC	AG	AG	TC	GG	GG	00	*B/O01*	B
76	1	2	GG	AC	AG	AG	TC	GG	GG	CC	*B/O01*	B
1	2	2	GG	AC	AG	AG	TC	00	GG	CC	*B/O01*	B
1	2	2	GG	AC	AG	00	TC	GG	GG	00	*B/O01*	B
3	2	2	GG	AC	00	AG	TC	GG	GG	00	*B/O01*	B
5	2	2	GG	AC	00	AG	TC	GG	GG	CC	*B/O01*	B
11	2	2	GG	AC	AG	AG	TC	GG	GG	00	*B/O01*	B
211	2	2	GG	AC	AG	AG	TC	GG	GG	CC	*B/O01*	B
2	2	2	GG	AC	AG	GG	TC	GG	GG	CC	*B/O01*	B
1	1	1	GG	AC	AG	AG	TC	00	GG	CC	*B/O01*	B
2	1	1	GG	AC	00	AG	TC	GG	GG	CC	*B/O01*	B
2	1	1	GG	AC	AG	AG	TC	GG	GG	00	*B/O01*	B
96	1	1	GG	AC	AG	AG	TC	GG	GG	CC	*B/O01*	B
1	1	1	GG	AC	AG	AG	TC	GG	TG	TC	*B/O01*	B
5	2	1	GG	AC	00	AG	TC	GG	GG	CC	*B/O01*	B
3	2	1	GG	AC	AG	AG	TC	GG	GG	00	*B/O01*	B
218	2	1	GG	AC	AG	AG	TC	GG	GG	CC	*B/O01*	B
1	2	1	GG	AC	AG	AG	TC	GG	TG	TC	*B/O01*	B
2	2	2	GG	AC	AG	AG	CC	AG	TG	TC	*B/O02*	B
1	1	2	GG	AC	00	AG	TC	AG	TG	TC	*B/O02*	B
55	1	2	GG	AC	AG	AG	TC	AG	TG	TC	*B/O02*	B

7	2	2	GG	AC	00	AG	TC	AG	TG	TC	*B/O02*	B
152	2	2	GG	AC	AG	AG	TC	AG	TG	TC	*B/O02*	B
2	2	2	GG	00	AG	AG	TC	AG	TG	TC	*B/O02*	B
1	1	1	GG	AC	AG	AG	TC	00	TG	TC	*B/O02*	B
1	1	1	GG	AC	00	AG	TC	AG	TG	TC	*B/O02*	B
79	1	1	GG	AC	AG	AG	TC	AG	TG	TC	*B/O02*	B
1	2	1	GG	AC	AG	00	TC	AG	TG	TC	*B/O02*	B
6	2	1	GG	AC	00	AG	TC	AG	TG	TC	*B/O02*	B
1	2	1	GG	AC	AG	AG	TC	AG	GG	00	*B/O02*	B
186	2	1	GG	AC	AG	AG	TC	AG	TG	TC	*B/O02*	B
2	2	1	GG	AC	AG	GG	TC	AG	TG	TC	*B/O02*	B
1	1	2	GG	CC	00	GG	CC	GG	GG	00	*O01/O01*	O
3	1	2	GG	CC	00	GG	CC	GG	GG	CC	*O01/O01*	O
6	1	2	GG	CC	GG	GG	CC	GG	GG	00	*O01/O01*	O
58	1	2	GG	CC	GG	GG	CC	GG	GG	CC	*O01/O01*	O
1	2	2	GG	CC	00	GG	CC	GG	GG	00	*O01/O01*	O
4	2	2	GG	CC	00	GG	CC	GG	GG	CC	*O01/O01*	O
4	2	2	GG	CC	GG	GG	CC	GG	GG	00	*O01/O01*	O
168	2	2	GG	CC	GG	GG	CC	GG	GG	CC	*O01/O01*	O
1	2	2	GG	CC	GG	GG	CC	GG	TG	TC	*O01/O01*	O
1	1	1	GG	CC	GG	GG	CC	00	GG	CC	*O01/O01*	O
2	1	1	GG	CC	00	GG	CC	GG	GG	CC	*O01/O01*	O
2	1	1	GG	CC	GG	GG	CC	GG	GG	00	*O01/O01*	O
83	1	1	GG	CC	GG	GG	CC	GG	GG	CC	*O01/O01*	O
3	2	1	GG	CC	00	GG	CC	GG	GG	CC	*O01/O01*	O
2	2	1	GG	CC	GG	GG	CC	GG	GG	00	*O01/O01*	O

143	2	1	GG	CC	GG	GG	CC	GG	GG	CC	*O01/O01*	O
1	1	1	0	CC	GG	GG	CC	AG	TG	TC	*O01/O02*	O
5	1	2	GG	CC	00	GG	CC	AG	TG	TC	*O01/O02*	O
80	1	2	GG	CC	GG	GG	CC	AG	TG	TC	*O01/O02*	O
1	2	2	GG	CC	GG	GG	CC	00	TG	TC	*O01/O02*	O
17	2	2	GG	CC	00	GG	CC	AG	TG	TC	*O01/O02*	O
1	2	2	GG	CC	GG	GG	CC	AG	GG	TC	*O01/O02*	O
255	2	2	GG	CC	GG	GG	CC	AG	TG	TC	*O01/O02*	O
1	2	2	GG	CC	GG	GG	CC	AG	TT	TT	*O01/O02*	O
2	1	1	GG	CC	00	GG	CC	AG	TG	TC	*O01/O02*	O
107	1	1	GG	CC	GG	GG	CC	AG	TG	TC	*O01/O02*	O
6	2	1	GG	CC	00	GG	CC	AG	TG	TC	*O01/O02*	O
241	2	1	GG	CC	GG	GG	CC	AG	TG	TC	*O01/O02*	O
3	1	2	GG	CC	00	GG	CC	AA	TT	TT	*O02/O02*	O
42	1	2	GG	CC	GG	GG	CC	AA	TT	TT	*O02/O02*	O
7	2	2	GG	CC	00	GG	CC	AA	TT	TT	*O02/O02*	O
1	2	2	GG	CC	GG	GG	CC	AA	00	00	*O02/O02*	O
85	2	2	GG	CC	GG	GG	CC	AA	TT	TT	*O02/O02*	O
49	1	1	GG	CC	GG	GG	CC	AA	TT	TT	*O02/O02*	O
2	1	1	GG	CC	00	GG	CC	AA	TT	TT	*O02/O02*	O
1	2	1	GG	CC	GG	GG	CC	00	TT	TT	*O02/O02*	O
2	2	1	GG	CC	00	GG	CC	AA	TT	TT	*O02/O02*	O
1	2	1	GG	CC	GG	GG	CC	AA	00	00	*O02/O02*	O
1	2	1	GG	CC	GG	GG	CC	AA	TG	TC	*O02/O02*	O
87	2	1	GG	CC	GG	GG	CC	AA	TT	TT	*O02/O02*	O
1	1	1	AG	AC	AG	AG	TC	AG	TG	TC	?	?

1	1	2	AG	AA	AA	AA	TT	GG	GG	CC	?	?
1	2	2	GG	AC	AG	AG	CC	GG	GG	CC	?	?
2	1	1	GG	AC	AG	AG	CC	GG	GG	CC	?	?
1	2	1	AG	AA	AA	AA	TT	GG	GG	CC	?	?
1	1	2	AA	CC	GG	GG	TC	AG	TG	TC	?	?
1	1	2	GG	CC	GG	GG	TC	AG	TG	TC	?	?
1	1	2	GG	CC	GG	GG	TC	GG	GG	CC	?	?
3	2	2	GG	CC	GG	GG	TC	AG	TG	TC	?	?
4	2	2	GG	AC	AG	AG	TT	GG	GG	CC	?	?
2	2	2	GG	CC	GG	GG	TC	GG	GG	CC	?	?
2	2	2	AG	CC	GG	GG	TT	GG	GG	CC	?	?
2	1	1	AA	CC	GG	GG	TC	AG	TG	TC	?	?
2	1	1	GG	CC	GG	GG	TC	AG	TG	TC	?	?
2	1	1	AG	CC	GG	GG	CC	GG	GG	CC	?	?
1	1	1	GG	CC	GG	GG	TC	GG	GG	CC	?	?
1	1	1	AG	CC	AG	GG	TT	GG	GG	CC	?	?
1	2	1	AG	CC	GG	GG	CC	AA	TT	TT	?	?
1	2	1	AA	CC	GG	GG	TC	AG	TG	TC	?	?
1	2	1	GG	CC	GG	GG	TC	AG	TG	TC	?	?
1	2	1	AG	AC	00	AG	TC	AG	TG	TC	?	?
1	2	1	AG	AC	AG	AG	TC	GG	GG	CC	?	?
3	2	1	GG	CC	GG	GG	TC	GG	GG	CC	?	?
1	2	1	AG	CC	GG	GG	TT	GG	GG	CC	?	?

Total number: 4531, including 2201 centenarians and 2330 mid-aged controls; 0 /?: unknown

Description of identification method:

ABO alleles

The identification methods of *ABO* alleles are as follows:

a) rs507666, AA: 2 *A* alleles, AG: 1 *A* allele, GG: no *A* allele

b) rs8176743, AA: 2 *B* alleles, AG: 1 *B* allele, GG: no *B* allele

c) rs8176746, AA: 2 *B* alleles, AC: 1 *B* allele, CC: no *B* allele

d) rs8176749, AA: 2 *B* alleles, AG: 1 *B* allele, GG: no *B* allele

e) rs687289, CC: 2 *O* alleles (*O01* and /or *O02*), TC: 1 *O* allele (*O01* or *O02*), TT: no *O* allele

f) rs512770, AA: 2 *O02* alleles, AG: 1 *O02* allele, GG: no *O02* allele

g) rs688976, TT: 2 *O02* alleles, TG: 1 *O02* allele, GG: no *O02* allele

h) rs549446, TT: 2 *O02* alleles, TC: 1 *O02* allele, CC: no *O02* allele

ABO genotypes

The identification methods of *ABO* genotypes are as follows:

a) rs507666: AA, rs8176743: GG, rs8176746: CC, rs8176749: GG, rs687289: TT, rs512770: GG, rs688976: GG and rs549446: CC for genotype *A/A*

b) rs507666: AG, rs8176743: GG, rs8176746: CC, rs8176749: GG, rs687289: TC, rs512770: GG, rs688976: GG and rs549446: CC for genotype *A/O01*

c) rs507666: AG, rs8176743: GG, rs8176746: CC, rs8176749: GG, rs687289: TC, rs512770: AG, rs688976: TG and rs549446: TC for genotype *A/O02*

d) rs507666: GG, rs8176743: AA, rs8176746: AA, rs8176749: AA, rs687289: TT, rs512770: GG, rs688976: GG and rs549446: CC for genotype *B/B*

e) rs507666: GG, rs8176743: AG, rs8176746: AC, rs8176749: AG, rs687289: TC, rs512770: GG, rs688976: GG and rs549446: CC for genotype *B/O01*

f) rs507666: GG, rs8176743: AG, rs8176746: AC, rs8176749: AG, rs687289: TC, rs512770: AG, rs688976: TG and rs549446: TC for genotype *B/O02*

g) rs507666: GG, rs8176743: GG, rs8176746: CC, rs8176749: GG, rs687289: CC, rs512770: GG, rs688976: GG and rs549446: CC for genotype *O01/O01*

h) rs507666: GG, rs8176743: GG, rs8176746: CC, rs8176749: GG, rs687289: CC, rs512770: AG, rs688976: TG and rs549446: TC for genotype *O01/O02*

i) rs507666: GG, rs8176743: GG, rs8176746: CC, rs8176749: GG, rs687289: CC, rs512770: AA, rs688976: TT and rs549446: TT for genotype *O02/O02*

j) rs507666: AG, rs8176743: AG, rs8176746: AC, rs8176749: AG, rs687289: TT, rs512770: GG, rs688976: GG and rs549446: CC for genotype *A/B*

It should be noted that if an error occurred in one or two of the three SNPs that represent allele *B*, the remaining one or two SNPs could represent allele *B* independently. The same treatment was used for allele *O02* and genotype analysis above. If the results of SNPs representing different alleles conflicted, they were marked as "unknown".
